# What Defines Different Modes of Snake Locomotion?

**DOI:** 10.1093/icb/icaa017

**Published:** 2020-04-09

**Authors:** Bruce C Jayne

**Affiliations:** Department of Biological Sciences, University of Cincinnati, Cincinnati, OH 45221-0006, USA

## Abstract

Animals move in diverse ways, as indicated in part by the wide variety of gaits and modes that have been described for vertebrate locomotion. Much variation in the gaits of limbed animals is associated with changing speed, whereas different modes of snake locomotion are often associated with moving on different surfaces. For several decades different types of snake locomotion have been categorized as one of four major modes: rectilinear, lateral undulation, sidewinding, and concertina. Recent empirical work shows that the scheme of four modes of snake locomotion is overly conservative. For example, during aquatic lateral undulation, the timing between muscle activity and lateral bending changes along the length of the snake, which is unlike terrestrial lateral undulation. The motor pattern used to prevent sagging while bridging gaps also suggests that arboreal lateral undulation on narrow surfaces or with a few discrete points of support has a different motor pattern than terrestrial lateral undulation when the entire length of the snake is supported. In all types of concertina locomotion, the distance from the head to the tail changes substantially as snakes alternately flex and then extend different portions of their body. However, snakes climbing cylinders with concertina exert forces medially to attain a purchase on the branch, whereas tunnels require pushing laterally to form an anchoring region. Furthermore, different motor patterns are used for these two types of concertina movement. Some snakes climb vertical cylinders with helical wrapping completely around the cylinder, whereas all other forms of concertina bend regions of the body alternately to the left and right. Current data support rectilinear locomotion and sidewinding as being distinct modes, whereas lateral undulation and concertina are best used for defining categories of gaits with some unifying similarities. Partly as a result of different motor patterns, I propose recognizing five and four distinct types of lateral undulation and concertina, respectively, resulting in a total of 11 distinct gaits previously recognized as only four.

## Introduction

Animals move in diverse ways depending on their body plan, the structure of the environment, and locomotor speed. The diversity of vertebrate locomotion is commonly categorized by defining different gaits or modes of locomotion for terrestrial limbed animals and aquatic and limbless species. At some level, the terms “gait” and “locomotor mode” can be used interchangeably (Webb 1994) given their common goal of identifying similarities and differences in patterns of movement, mechanics, or neural control. Herein, I will review the locomotion of snakes, in part as a model system to gain general insights into the merits of using different criteria to define gaits.

Some definitions of gaits use quantitative criteria and split a continuum of variation into convenient categories. A classic example of this approach for limbed locomotion is partitioning a bivariate plot of the relative duration of foot contact (duty factor) and the phase lag between an ipsilateral pair of limbs (Hildebrand 1976). Although some portions of this kinematic space have discontinuities when duty factor is low, different gaits are also recognized in regions that lack any apparent discontinuities. Several modes of undulatory swimming also divide a continuum of variation from the extremes of undulating the whole body (anguilliform) or a highly restricted region (thuniform) as well as some intermediate gaits such as carangiform and sub-carangiform swimming (Webb and Blake 1985).

In some cases, quantitative variation creates qualitative differences. For example, the continuous variation in both duty factor and the phase lag between limb movements can give rise to a different sequence in the number of feet that simultaneously contact the ground (Hildebrand 1976). In other cases, clear qualitative (presence or absence) traits may differentiate gaits as when fish use different structures such as the pectoral, dorsal, or anal fins for different gaits (Webb and Blake 1985). Of course, even when different structures are used, quantitative variation in the amplitude and frequency of movement also commonly occurs for a given structure that contributes to propulsion.

For a given species, much of the variation in the gaits of limbed animals is associated with different speeds of locomotion, and many species of fish also have a regular progression of different modes of swimming as speed increases. Although some variation in the gaits of snakes is associated with variation in speed, the shape and mechanical properties of surfaces are dominant factors influencing the use of different modes of locomotion (Mosauer 1932; Gray 1946; Gans 1962; Jayne 1986).

For several decades workers have identified four major modes of snake locomotion: rectilinear, lateral undulation, sidewinding, and concertina locomotion (Mosauer 1932; Gray 1946; Lissmann 1950; Gans 1962; Jayne 1986). In contrast to the latter three modes, which use vertebral bending to general propulsive forces, movement of the skin relative to the underlying skeleton propels snakes during rectilinear locomotion (Lissmann 1950). During lateral undulation all points along the length of the snake move simultaneously and have sliding contact with the environment as regions bending to the left and right propagate along the entire length of the snake (Gray 1946). Sidewinding also involves bending from side to side that is posteriorly propagated, but snakes using this mode also arch their back to lift the body between regions of static contact with the ground (Mosauer 1932). During concertina locomotion snakes have both sliding and static contact, and the head-to-tail distance changes as a convoluted region of the body provides a static anchoring region with subsequent straightening that slides the body forward (Gray 1946). These general verbal definitions of snake gaits, based on easily recognizable qualitative traits describing movement but not motor pattern, have been widely adopted, including in very recent literature.

Prior to the mid-1980s, quantitative kinematic data for the locomotion of snakes were sparse, and motor patterns had not been determined experimentally. The only information on the energetics of snake locomotion was a preliminary study that was never published in its entirety (Chodrow and Taylor 1973). Furthermore, unlike the complex structural variation that snakes often encounter naturally, most laboratory studies have understandably focused on using simpler conditions to elicit different modes of snake locomotion and studying movement mainly in a horizontal plane.

Since the mid-1980s, electromyography has been used to determine the muscular mechanisms of all four of the previously recognized snake locomotor modes (Jayne 1988b, 1988c; Gasc et al. 1989; Moon and Gans 1998; Newman and Jayne 2018). The energetic costs have also been determined for terrestrial lateral undulation, sidewinding, and concertina locomotion (Walton et al. 1990; Secor et al. 1992). More recently, studies have gained additional insights from a greater emphasis on more complex three-dimensional movements including locomotion on the cylindrical and discontinuous (gaps) surfaces that are characteristic of arboreal habitats. Instead of an exhaustive review of all of the literature regarding snake locomotion, this review focuses primarily on the more recent advances that facilitate differentiating gaits.

## Relevant anatomy

**Fig. 1:**
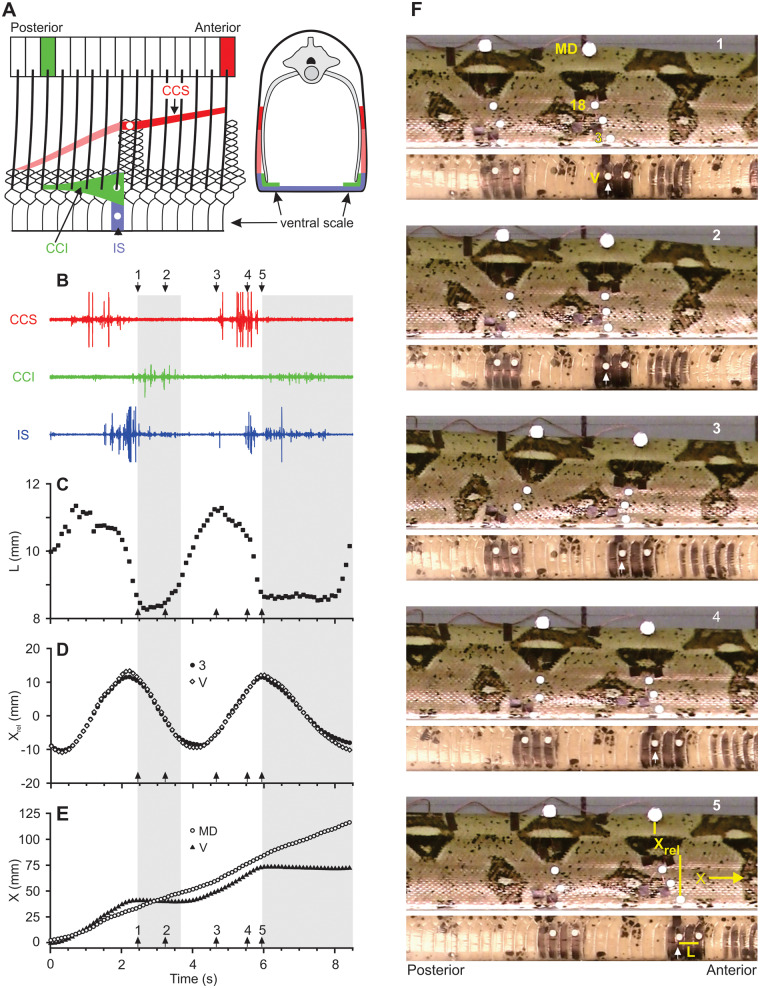
Muscles and movements involved in the rectilinear locomotion of a boa constrictor. (**A**) Schematic views of muscles for which red, green, and blue indicate the contractile tissue of the CCS, CCI, and interscutalis (IS) muscles, respectively. The lighter shade of red indicates less-mobile fibers of the CCS that are firmly connected to the skin along their entire length. The left image is an internal view of the left side of the snake, and the right image is a cross-section. The thick oblique lines indicate ribs, and the colored rectangles indicate the vertebrae of origin for the costocutaneous muscles that act on a single longitudinal location. Unlike the intact snake, the ventral skin is peeled back into a parasagittal plane so that the mid-ventral line is at the bottom of the figure. White circles indicate approximate locations for recording electrodes. As shown at right, the entire width of the ventral scale and the first two dorsal scale rows on both sides of the snake usually contact the ground. EMGs of muscle activity (**B**) and corresponding kinematics including the length of the ventral skin (**C**) and the longitudinal positions of landmarks (MD = mid-dorsal, 3 = third dorsal scale and V = ventral scale) relative to a mid-dorsal point (**D**) and a fixed frame of reference (**E**). Values of *X*_rel_ for the ventral (V) and third dorsal (3) scales have been standardized to a mean value of 0 (D).The gray areas in (B–**E**) indicate static contact between the ventral skin and the ground as well as some slight backward slipping. Video images of the right side of the snake (top) and ventral scales (bottom) of the snake for the same data as shown in (B–E) (**F**). Adapted from Newman and Jayne (2018). See supplementary video at https://www.youtube.com/watch?v=wwHkAMo-Mj0.

Compared to many other groups of amniotic vertebrates, vast areas of skin along the snake’s body are vitally important for transmitting locomotor forces (Cundall 1987). Snakes usually have overlapping scales that are thicker and stiffer than the skin in the hinge regions between scales (Jayne 1988a). Consequently, the skin can often stretch substantially without exposing the hinge region to the surfaces upon which the snake is crawling. The mid-ventral scales of scolecophidians are as small as the dorsal scales, whereas those of henophidians are substantially wider but not always wide enough to preclude some of the more ventrally located dorsal scales from contacting the ground (Fig. 1A and F). Except for some highly specialized aquatic species (Voris 1977), nearly all caenophidians have wide ventral scales (gastrosteges) that encompass the entire contact area with the ground. Unlike the more variable dorsal scales (i.e., all scales on the body other than the gastrosteges; Pough et al. 2016), the ventral scales usually are macroscopically more uniformly smooth, but the microscopic structure does vary and may contribute to directionally dependent frictional resistance (Hu et al. 2009).

Although the mid-dorsal skin of snakes attaches firmly to the vertebrae, the skin along the belly and lateral to the ribs can move substantially relative to the skeleton, and several muscles attach to the skin in this region. Unlike limbless lizards, snakes have costocutaneous inferior (CCI) and superior (CCS) muscles that connect the ventral and ventro-lateral skin to the ribs (Fig. 1A). Hence, costocutaneous muscles are a derived trait for snakes. A commonly overlooked fact is that costocutaneous muscles and mobile skin are absent in the tails of snakes, which can range from <5% to >40% of their total length in some burrowing and slender arboreal species, respectively (Sheehy et al. 2016). Consequently, the portion of the snake that can use rectilinear locomotion is highly variable among species, and species that are highly reliant on rectilinear locomotion may benefit from having rather short tails. Snakes also have many muscles intrinsic to the skin (Buffa 1904), one of the largest of which is the interscutalis (IS) muscle with longitudinally oriented fibers that extend between adjacent ventral scales (Fig. 1A).

**Fig. 2:**
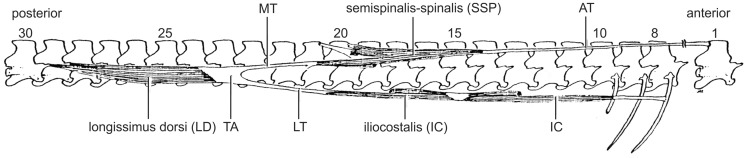
Simplified right, lateral view of major epaxial muscles of a water snake (*Nerodia fasciata pictiventris*). AT, anterior tendon of the SSP; TA, tendinous arch; MT, medial tendon; LT, lateral tendons.

Of the several additional axial muscles of snakes, the three largest expaxial muscles commonly comprise more than half of the total cross-sectional area of the axial muscles (Hoefer and Jayne 2013) and from dorsal to ventral these are the semispinalis-spinalis (SSP), longissimus dorsi (LD), and the iliocostalis (IC; Fig. 2). The longitudinal columns formed by these muscles consist of individual segments that have a 1:1 correspondence with the numbers of vertebrae. Individual segments of major epaxial muscles of snakes span several vertebrae, partly as a result of having long tendons, and depending upon the species, some of them have tendinous connections to each other (Fig. 2). Besides these three major muscles, many additional muscles extend between vertebrae, from the vertebrae to ribs and between ribs, their activity during locomotion has not be determined using electromyography.

## Rectilinear locomotion

Rectilinear locomotion uses different anatomical structures for propulsion than all other modes of snake locomotion. The ventral and ventro-lateral skin of snakes during rectilinear locomotion periodically shortens and lengthens while this portion of the skin oscillates longitudinally relative to the underlying skeleton (Lissmann 1950). Although the body need not be straight during this mode, snakes using rectilinear locomotion are able to move while the body is perfectly straight. Despite superficial appearances to the contrary, the ribs during rectilinear locomotion are immobile rather than contributing to some of the movement of the skin that covers the ribs (Lissmann 1950; Capano and Brainerd 2020). Lissmann hypothesized that the CCS and CCI move the skin anteriorly and posteriorly relative to the skeleton, respectively, and that the interscutalis propels the snake forward.

Electromyographic experiments (Newman and Jayne 2018) support Lissmann’s hypotheses regarding the antagonistic functions of the CCS and CCI (Fig. 1B and D). However, electromyographic recordings (EMGs) of a large, intrinsic, ventral skin muscle (interscutalis) are not consistent with the function of directly propelling the snake. Instead, initial concentric activity of the interscutalis (Fig. 1B) correlates first with shortening the ventral skin, and then isometric activity keeps the skin shortened during static contact with the ground as the CCI pulls the skeleton forward over this region (Fig. 1). Hence, the intrinsic skin musculature provides a mechanism for modulating the effective stiffness of the skin, which plays a vital role for the transmission of forces.

Many clades of lizards convergently evolved elongate limbless bodies, but apparently none have costocutaneous or any other muscles that would permit rectilinear locomotion. However, amphisbaenians (except for *Bipes*) are another group of limbless squamate reptiles, and in tunnels they can propel themselves forwards and backwards by moving their skin (Gans 1974). I presume that this involves anteriorly propagated EMGs and is facilitated by a lack of overlapping scales. For snakes, only forward rectilinear locomotion with posteriorly propagated EMGs has been observed.

## Lateral undulation

**Fig. 3:**
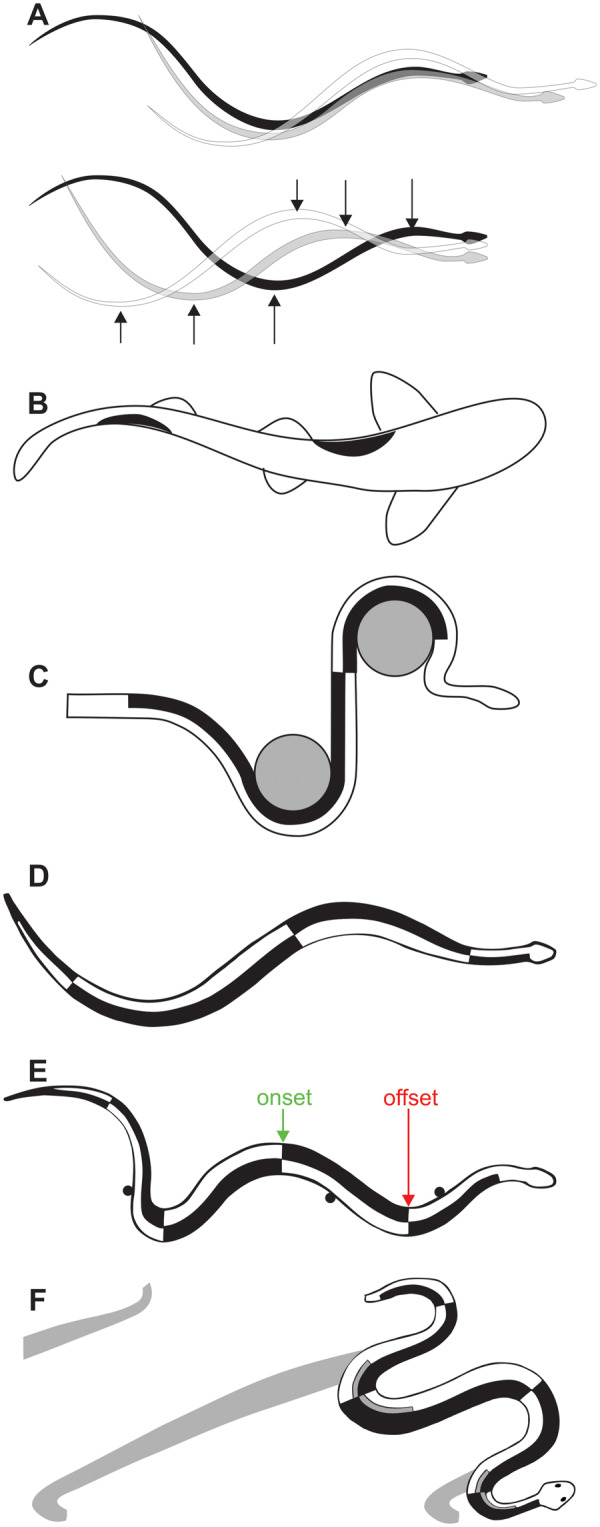
Dorsal views of locomotion with posterior propagation of lateral bending. (**A**) Swimming of a juvenile water snake (*N. fasciata pictiventris*). The images traced from films are at intervals of 0.4 s. The top three images are relative to a fixed frame of reference, whereas the bottom three images have been superimposed so that the snout is at the same longitudinal location. The arrows show homologous waves of bending on the left and right sides being propagated posteriorly. Hypothesized (**B, C**) and observed (**D–F**) axial muscle activity. Areas shaded back within the animal indicate muscle activity. (**B**) Gans (1974, Figure 3–9) used images of a swimming dogfish from Gray (1933) to illustrate his hypothesis that muscles were active on the concave sides of the fish where muscle fibers would be shorter than their resting length. (**C**) For a hypothetical snake crawling past two pegs, Gray and Lissmann (1950, Figure 11) also hypothesized activity of muscles in the concave regions of the snake. Unlike this illustration, however, when snakes crawl past pegs, the pegs are usually midway between rather than at the crests of the waves of bending. (**D–F**) Schematic summaries of EMGs of the major epaxial muscles of snakes. The outlines of the snakes were traced from filmed images, and the areas shaded black within the snake indicate simultaneous ipsilateral activity of the SSP-SP, LD, and IC muscles (Fig. 2). Because EMGs are propagated posteriorly, the posterior and anterior edges of the blackened areas indicate EMG onset and offset, respectively. Water snakes (*N. fasciata pictiventris*) during swimming (**D**) and crawling on a horizontal surface with pegs (**E**) based on Jayne (1988a, Figure 18). The timing of muscle activity relative to bending differs between swimming and terrestrial lateral undulation as well as differing along the length of the swimming snake. **(F)** Sidewinding of a sidewinder rattlesnake (*Crotalus cerastes*) based on Jayne (1988c). The gray areas within the snake indicate activity of the SSP without attendant activity of ipsilateral segments of the LD and IC muscles at the same longitudinal location. The gray areas outside of the snake indicate past regions of static contact with the ground. The bilateral activity of the SSP is associated with arching the back of the snake and lifting it off the ground.

Using posteriorly propagated waves of lateral bending to generate propulsive forces is likely a symplesiomorphy in vertebrates given its wide-spread presence in cephalochordates (Stokes 1997), lampreys, cartilaginous and bony fishes (Cohen 1988), and aquatic amphibians (Frolich and Biewener 1992). Consequently, a common and long-lasting presumption was that snakes performing lateral undulation in water and on land were using essentially the same type of primitive locomotion. For example, prior to electromyographic studies, the undulatory swimming of fishes was often used as a model for the lateral undulation of snakes (Gray 1968; Gans 1974). Another common assumption at this time was that axial muscles were active in the locations where their fibers were shorter than resting length (Fig. 3B;Gans 1985); hence, the muscles would be active in the laterally concave regions of the animal (Fig. 3B and C).

Although aquatic lateral undulation and terrestrial lateral undulation on horizontal substrates are different, they do share the following qualitative traits. First, regions of lateral bending to the left and right are propagated posteriorly along the entire length of the animal. Second, electromyographic studies of swimming in fishes with elongate shape (lamprey) and generalized shape (trout; Williams et al. 1989) and aquatic and terrestrial lateral undulation of snakes (Jayne 1988b) have all found muscle activity that is propagated from head to tail, and at a given longitudinal location muscle activity is unilateral and alternates between the left and right sides (Fig. 3D and E). Third, during both terrestrial and aquatic lateral undulation of snakes, the ipsilateral activity of the three largest epaxial muscle (SSP, LD, and IC) at a given longitudinal location is also nearly synchronous (Jayne 1988b; Gasc et al. 1989; Moon and Gans 1998).

The following quantitative differences occur between the aquatic and terrestrial lateral undulation of snakes. In swimming snakes both the amplitude and wavelength of the waves of bending increase regularly from anterior to posterior (Fig. 3A), and the speed of mechanical wave propagation exceeds the forward speed of the snake. Consequently, for both of these reasons, different points along the length of a swimming snake follow substantially different paths (Fig. 3A). By contrast, during terrestrial lateral undulation, solid objects in the environment often prevent slipping, and the speeds of wave propagation and forward movement are the same as all of the points along the length of the snake more or less follow the same path. In terrestrial lateral undulation, the speed of propagation of muscle activity also matches that of the mechanical wave of bending, whereas, muscle activity is propagated faster than the mechanical wave of bending during swimming (Jayne 1988b). Thus, terrestrial lateral undulation has concentric muscle activity where the constant phase relationship with lateral bending results in onsets and offsets that are on the side of the body where it is maximally convex and maximally concave, respectively (Fig. 3E). By contrast, in swimming snakes, the phase relationship between bending and muscle activity shifts progressively along the length of the snake so that a substantial amount of posterior (eccentric) muscle activity occurs as the muscle fibers lengthen (Fig. 3E). These relationships between motor pattern and bending are most easily recognized by noting the locations of the transition between muscle activity on the left and right sides (Fig. 3D and E). A longitudinal phase shift between EMGs and bending similar to that of swimming snakes is effectively universal in the undulatory swimming of fishes, and the resulting eccentric muscle activity probably stiffens the posterior body as it pushes against the water (Wardle et al. 1995).

Some species of sea snake, such as *Hydrophis platurus* (Hydrophiinae, Elapidae), are equally adept at swimming forward and backward using lateral undulations (Heatwole 1999). I have never observed backward terrestrial lateral undulation in a snake, which would probably be impeded by the backward-pointing scales of generalized snakes. Even though being in water would seem to negate this problem, I have never observed backward swimming in a wide variety of colubrid and henophidian snakes. However, I recently observed a file snake *Acrochordus granulatus* (Acrochordidae) swimming backward (B.C. Jayne, unpublished data). File snakes and true sea snakes are phylogenetically very distant (Figueroa et al. 2016), which strongly suggests that the ability to reverse the direction of a propagated epaxial motor pattern may be a derived trait that has only rarely evolved convergently in some highly specialized lineages of snakes. Another derived trait of sea snakes (and *A. granulatus*), the paddle-shaped tail, emphasizes the importance of the entire height of the snake for pushing against water to provide propulsion, whereas nearly all locomotion of snakes on solid surfaces uses only a fraction of the entire surface area to push against the environment.

**Fig. 4:**
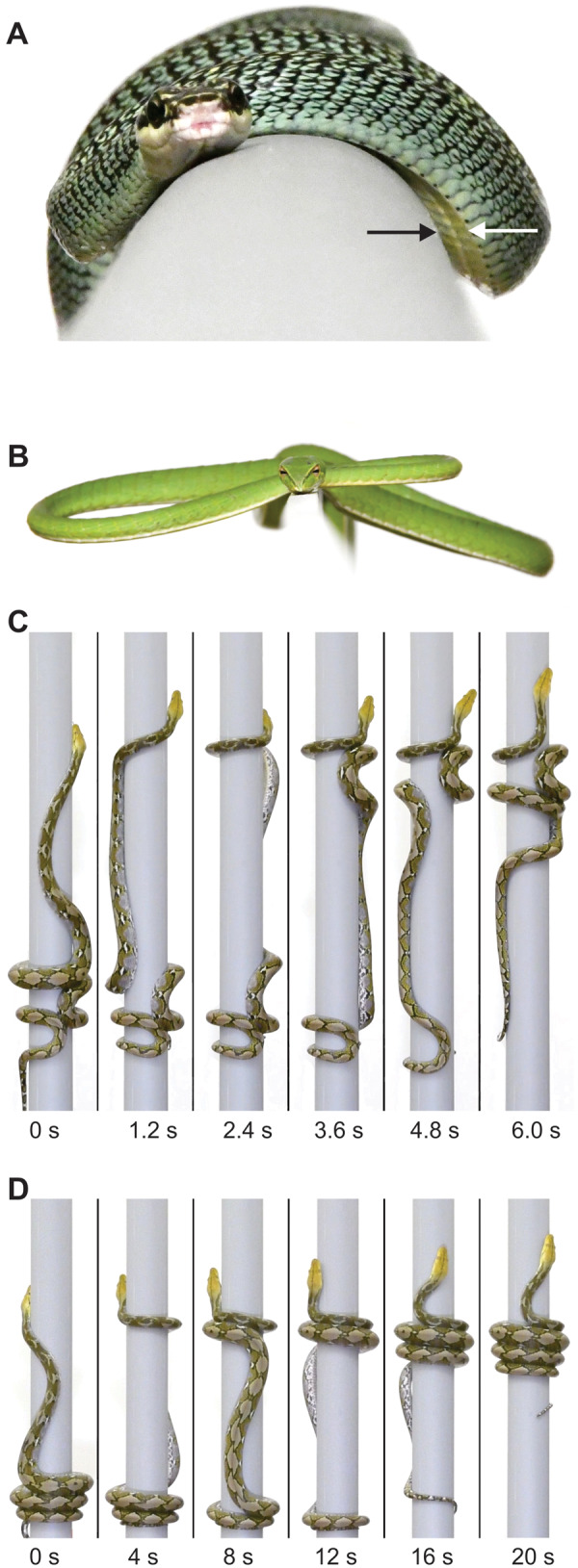
Mechanisms for preventing slipping and falling during arboreal locomotion. (**A**) Lateral undulation of a highly arboreal gliding snake (*Chrysopelea ornata*, total length, *L* = 112 cm) on a smooth horizontal cylinder with a diameter of 5 cm. The snake can modulate the sharpness of the longitudinal keels (indicated by arrows) that are near the lateral margins of the ventral scales. The leading edge of the snake (white arrow) often does not contact the surface, whereas contact of the sharp keel on the trailing edge of the lateral bend (black arrow) occurs and appears to reduce backward slipping. See supplementary videos at https://www.youtube.com/watch? v=KT2kcqvhPnQ and https://www.youtube.com/watch? v=ZnSHVAjULyI. (**B**) A Southeast Asian vine snake (*Ahaetulla prasina L* = 113 cm) balancing on a flat surface only 6 mm wide. (**C, D**) Gripping during concertina locomotion of a reticulated python (*P. reticulatus*, *L* = 197 cm) climbing a vertical cylinder with a 9 cm diameter. The python may use either the more common behavior of bending alternately to the left and right (C) or wrapping helically with nearly all of the body (D) to grip the cylinder. All of the cylinders were covered with gaffer’s tape (Shurtape P-665).

A longstanding and logical presumption is that lateral undulation on solid surfaces requires some surfaces against which the sides of the snake push so that the posterior propagation of a bend can generate reactive forces that are oriented anterio-medially. Having more than one such point of force application allows the left and right components to cancel each other out and generate a resultant propulsive force vector in the overall direction of forward movement (Gray and Lissmann 1950). Another long-held view was that a minimum of three discrete points of force application are required for stable lateral undulation (Gans 1974). However, subsequent experiments have shown this not to be the case. For example, some arboreal species of snakes perform steady lateral undulation with fewer than three discrete points of force application (Jayne et al. 2013). Furthermore, some species of snakes can perform lateral undulation on smooth solid surfaces lacking any macroscopic projections that are large enough to contact the sides of the snake (Hu et al. 2009; Jayne et al. 2015). Perhaps directionally dependent frictional properties of snake skin contribute to this ability (Hu et al. 2009), but some arboreal species can also make a keel (Fig. 4A) that may reduce slipping on surfaces that superficially seem smooth and ill-suited for lateral undulation (Jayne et al. 2015). Snakes such as *Chrysopelea* can modulate the sharpness of the keel (Fig. 4A), which suggests they use some different muscles with a different motor pattern (and mode of locomotion) than generalized lateral undulation on solid surfaces.

On cylindrical surfaces with shallower inclines, sideways toppling becomes more problematic, but many snakes adeptly maintain their balance on surfaces that are narrow relative to their size (Fig. 4B). However, the speed and ease of arboreal lateral undulation can also be enhanced either by crawling on multiple branches simultaneously or crawling on branches with secondary branches that can prevent sideways toppling (Astley and Jayne 2009; Jayne et al. 2015). The uneven distribution of weight after snakes consume a large meal can also compromise their ability to balance during arboreal locomotion (Crotty and Jayne 2015).

Unlike terrestrial lateral undulation, the unsupported regions of snakes performing arboreal lateral undulation may sag considerably unless some active mechanism prevents this (Fig. 4B). Electromyographic data are not available for snakes performing arboreal lateral undulation. However, if snakes use a motor pattern similar to that which prevents sagging during gap bridging (Jorgensen and Jayne 2017), then the motor pattern during arboreal lateral undulation would differ from that of generalized terrestrial lateral undulation as a result of having prolonged bilateral activity of the SSP where needed to prevent excessive sagging.

**Fig. 5.**
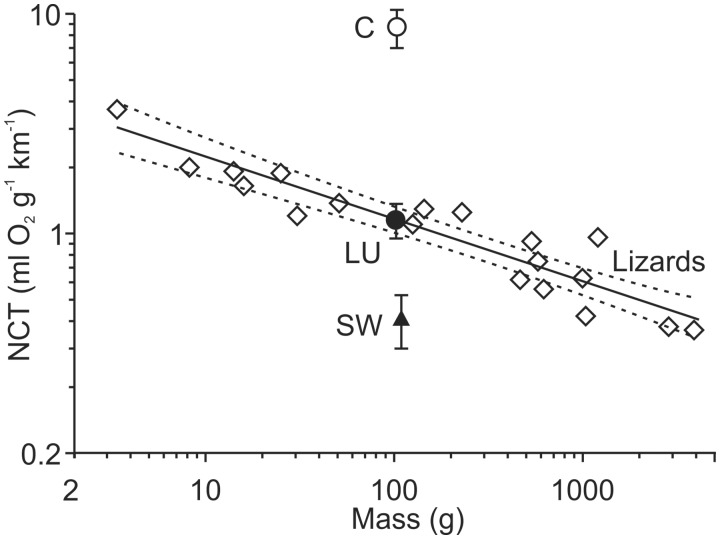
Net cost of transport (NCT) for the terrestrial locomotion of limbed lizards and snakes adapted from (Secor et al. 1992). The values of NCT (±1 SE) for snakes are for a black racer (Walton et al. 1990) performing terrestrial lateral undulation (black circle) and concertina within a horizontal tunnel (white circle) and for sidewinding rattlesnake (triangle) on a treadmill (Secor et al. 1992). Diamonds are values for different species of lizards (John-Alder et al. 1986), for which a collective scaling equation (±95% CL) is shown. Note that the NCT of terrestrial lateral undulation of a snake is statistically indistinguishable from that of a lizard with similar mass, and NCT differs radically among different modes of snake locomotion.

In summary, I favor using the term “lateral undulation” as an overarching category that encompasses five distinct modes, all of which involve alternating left and right bends that are propagated along the entire snake as all points along the snake move simultaneously and have sliding contact where they touch their surroundings. I propose using the following five terms to recognize these distinct types of lateral undulation: (1) forward aquatic lateral undulation (Fig. 3A and D), (2) backward aquatic lateral undulation, (3) terrestrial lateral undulation (Fig. 3E), (4) lateral undulation with a ventrolateral keel (Fig. 4A), and (5) arboreal lateral undulation (with active prevention of sagging in unsupported regions as in Fig. 4B).

## Sidewinding

At first glance sidewinding seems radically different from terrestrial lateral undulation, partly because it allows movement on flat smooth uncluttered surfaces upon which terrestrial lateral undulation is difficult or impossible. Movements of sidewinding differ from terrestrial lateral undulation by the following traits: (1) some parts of the snake have static contact with the ground, (2) all points along the length of the snake follow distinctly different but nearly parallel paths, (3) regions of static contact create disconnected tracks that are parallel to each other and oblique to the overall direction of travel (Fig. 3F), and (4) posterior segments of the snake are consistently in front of more anterior segments within a region of static contact (Fig. 3F). The net cost of transport of sidewinding locomotion (Secor et al. 1992) is also substantially less than that of a snake performing terrestrial lateral undulation (Fig. 5; Walton et al. 1990).

Sidewinding and terrestrial lateral undulation have the following major similarities (Jayne 1988c). First, in both of these modes the crest of each wave of lateral bending travels posteriorly along the entire length of the snake. Second, in both of these modes, the LD and IC at a particular longitudinal location have nearly synchronous activity that is alternating, unilateral and posteriorly propagated. Finally, in both of these modes, the onset and offset of both LD and IC activities occur in regions that are maximally convex and maximally concave, respectively (Fig. 3E and F).

Two features of the axial motor pattern of sidewinding differ from terrestrial lateral undulation (Jayne 1988c). First, the activity of the SSP in some regions is decoupled from that of the ipsilateral segments of the LD and IC at the same longitudinal location (Fig. 3F). Second, this novel SSP activity occurs together with that of contralateral segments of the SSP at the same longitudinal location, and this bilateral activity arches the snake’s back and lifts it up off the ground in between the regions of static contact with the ground (Fig. 3F).

## Concertina

**Fig. 6:**
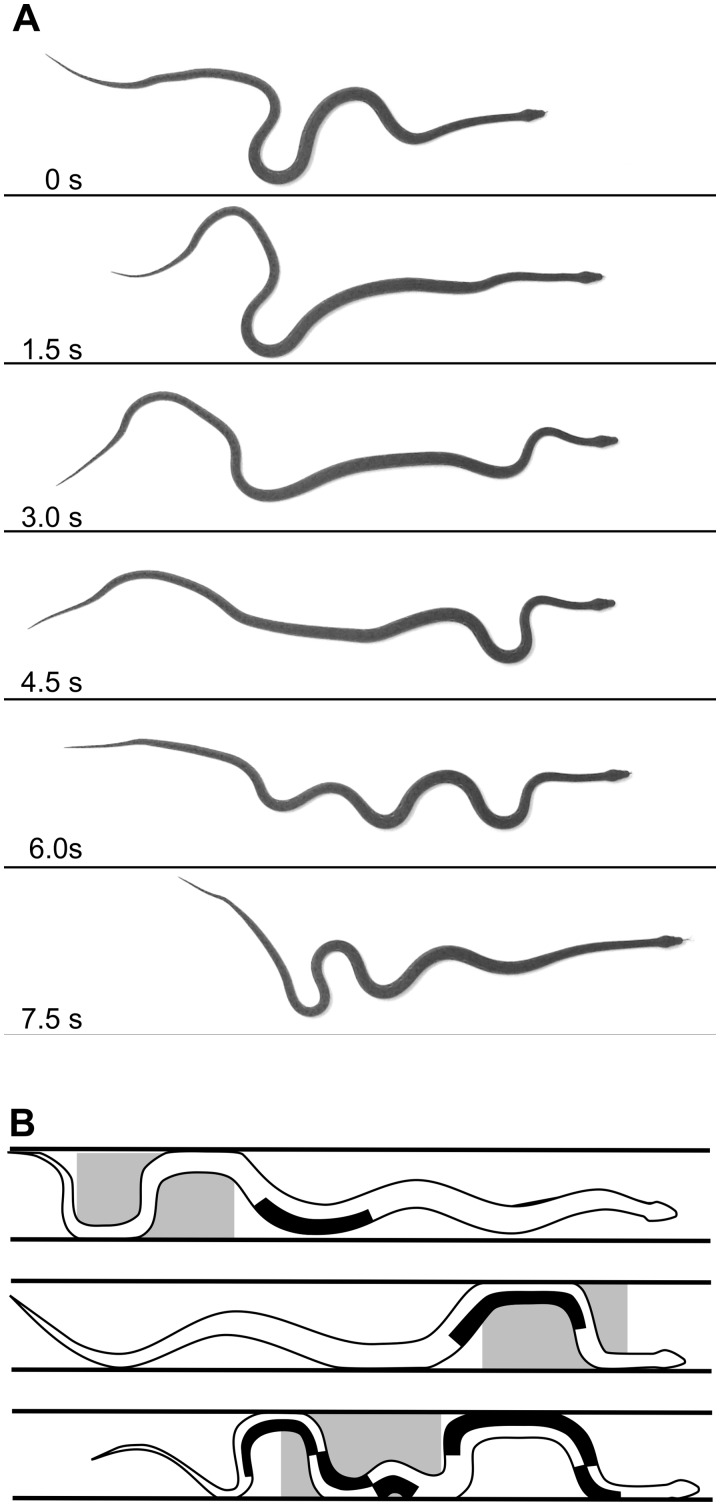
Concertina locomotion on flat horizontal surfaces. (**A**) Video images of a reticulated python (*P. reticulatus*, *L* = 197 cm). The flat, smooth surface was a sheet of Sintra^®^Board, which had coefficients of forward and backward static friction of 0.17 and 0.22, respectively. (**B**) Schematic summary of EMGs for a Florida banded water snake (*N. fasciata pictiventris*, *L* = 100 cm) in a flat tunnel 10 cm wide based on Jayne (1988c). The outlines of the snake were traced from filmed images, and the areas shaded black indicate simultaneous activity of the ipsilateral SSP, LD, and IC muscle segments. The gray regions indicate static contact between the snake and surfaces.

**Fig. 7:**
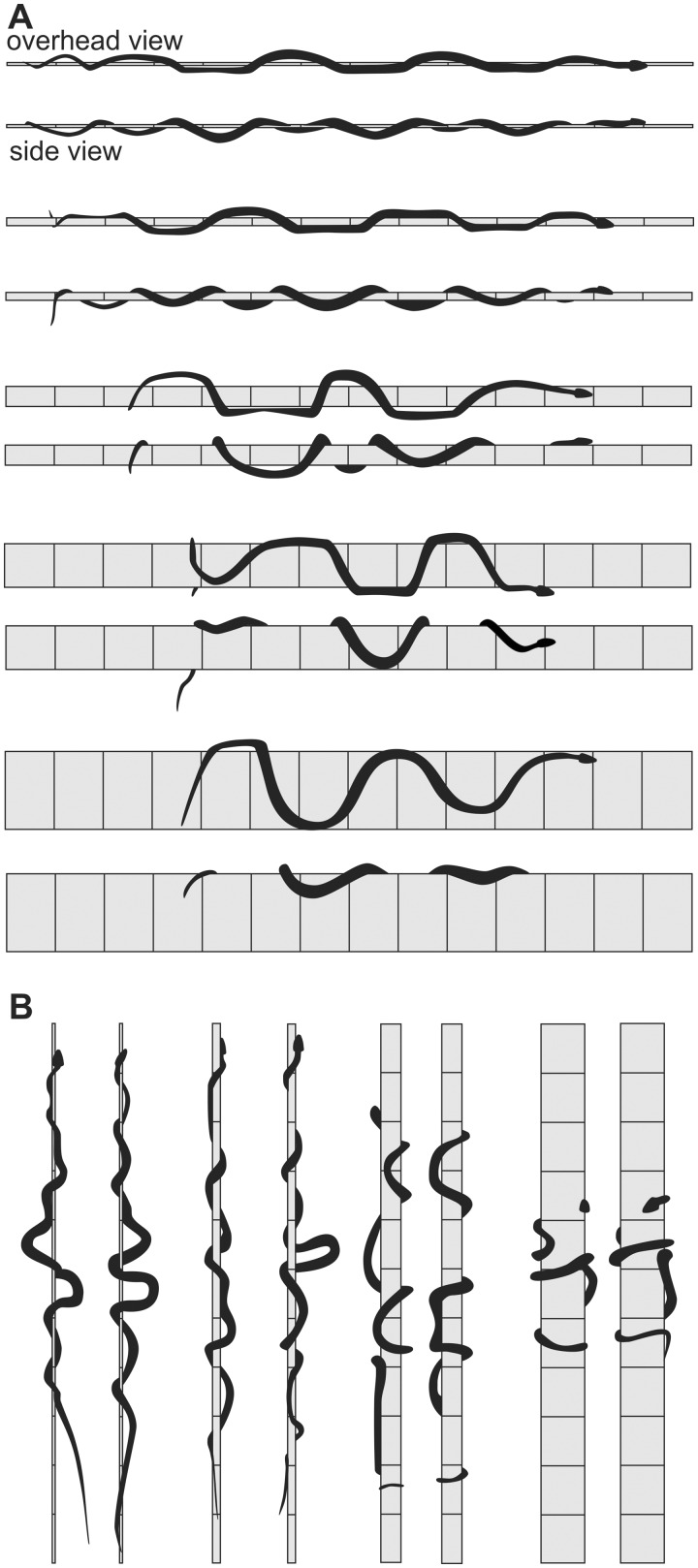
Arboreal locomotion of a brown tree snake (*B. irregularis*, approximate *L* = 135 cm) on smooth cylinders based on (Jayne and Byrnes 2015). (**A**) Lateral undulation on horizontal cylinders. (**B**) Concertina locomotion climbing up a vertical cylinder. For each combination of cylinder diameter and incline, a pair of images shows the overhead and lateral views. All of the cylinders were covered with duct tape, and the reference lines on the cylinder indicate 10 cm intervals. The diameters of the cylinders were 0.6, 1.6, 4.1, 8.9, and 15.9 cm. Note the varying extent to which the body of the snake encircles the cylinder and conforms to its shape.

Concertina locomotion may be performed on flat, smooth horizontal surfaces, within the confines of tunnels and on cylindrical surfaces such as branches (Wiedemann 1932; Gray 1946; Gans 1974; Astley and Jayne 2009). Snakes performing concertina locomotion do not propagate a homologous bend posteriorly along their entire length (Figs. 4C and D and 6), which is unlike both sidewinding and lateral undulation. However, in all types of concertina locomotion, the convolutions that anchor the snake via static contact are created from anterior to posterior (Figs. 4C and D and 6). With one notable exception (Fig. 4D), all types of concertina use alternating bends to the left and right to establish anchoring regions of static contact (Figs. 4C and D, 6, and 7B).

Previous reviews of snake locomotion (Gray 1946; Edwards 1985; Gans 1985) often mentioned flat-surface concertina despite scant empirical data (Lillywhite 2014). In the original description of this mode (Wiedemann 1932) and in many subsequent articles often lacking mention of a particular species (Gray 1946; Edwards 1985; Gans 1985; Lillywhite 2014), the illustrations appear to be extremely schematic rather than based directly on experimental data. Hence, I recently videotaped 17 bouts of movement from three reticulated pythons (*Python reticulatus*, total lengths = 179–197 cm) on a flat surface in the laboratory (Fig. 6A), and I observed three key differences with an oft-repeated schematic illustration (Figure 8 in Gray 1946). First, the snakes I observed were never transiently straight along their entire length. Second, the regions of bending formed and straightened in a progression from anterior to posterior rather than happening simultaneously. Third, substantial backward slipping often occurred in the posterior region of the snake as more anterior regions were sliding forward (Fig. 6A). Importantly, unlike all other types of concertina, during flat-surface concertina, all normal force contributing to static friction in the anchoring region arises passively from the snake’s weight.

In contrast to flat-surface concertina, concertina locomotion within tunnels is very well studied, including its energetics and motor pattern. The energetic cost of this mode (Walton et al. 1990) greatly exceeds that of both terrestrial lateral undulation and sidewinding (Fig. 5), and the difficulty of performing this mode is affected greatly by the tunnel width (Jayne and Davis 1991). During tunnel concertina locomotion as in terrestrial and aquatic lateral undulation, at a given longitudinal location, EMGs of the ipsilateral SSP, LD, and IC segments are nearly synchronous and unilateral (Fig. 6B;Jayne 1988c). Concentric muscle activity (during fiber shortening) creates the convoluted anchoring regions, and subsequent prolonged isometric activity, often in the concave regions, maintains the lateral pressure against the sides of the tunnel (Fig. 6B). Much of the activity in the sliding region anterior to an anchoring region is on the convex sides as they straighten and propel the snake forward (Fig. 6B). Unlike all other previously discussed axial motor patterns during snake locomotion, this one is not propagated along the entire length of the snake.

Arboreal concertina locomotion on cylindrical surfaces (Figs. 4C and D and 7B), such as branches, differs from both flat-surface concertina and tunnel concertina because the snakes use active ventral flexion to press medially and form a static grip with the cylinder (Byrnes and Jayne 2014). Diverse species of snakes accomplish this most commonly via a series of alternating bends to the left and right (Figs. 4C and 7B). As in tunnel concertina, increased width of the surface increases the amplitude and wavelength of the bending regions for snakes on cylinders (Fig. 7B). Unlike tunnel concertina, the lateral displacement of the snake on a cylinder is not constrained, and considerable variation occurs among different species and among different diameters of cylinders for the extent to which the shape of the snake conforms to that of the cylinder (Fig. 7B). Unlike most lateral undulation on cylinders (Fig. 7A), the body of the snake during concertina usually makes at least a 180° arc around the circumference of the cylinder (Fig. 7B). In some cases a successive pair of alternating bends overlaps so much that the cylinder is completely encircled by a continuous region of the snake’s body (Fig. 7B, largest diameter).

**Fig. 8:**
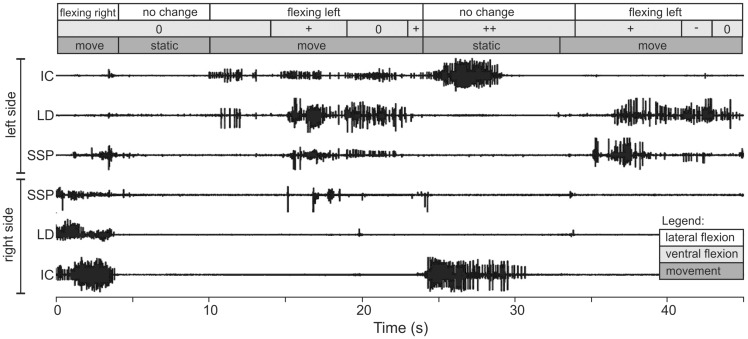
EMGs from the epaxial muscles of a boa constrictor (*Boa constrictor*, approximate *L* = 132 cm) using arboreal concertina with alternating loops to climb a smooth cylinder (diameter = 5 cm) inclined 45°. The major bilateral activity of the IC without attendant activity of the either the SSP or LD (25–30 s) occurred when this portion of the body was perpendicular to the long axis of the cylinder and forming a static grip via ventral flexion. Such bilateral activity has not been observed for concertina in horizontal tunnels with flat floors and flat sides. The boxes above the EMGs are rough estimates of the time course of kinematic events. For ventral flexion of the vertebrae, ++, +, 0, and - indicate conspicuous ventral flexion, slight ventral flexion, little discernable flexion, and slight dorsal flexion, respectively. These data are unpublished observations from Jayne and Newman.

In some ways, the epaxial motor patterns during arboreal concertina locomotion with alternating bends on a cylinder (Fig. 8) and in a tunnel differ more from each other than the differences between the sidewinding and terrestrial lateral undulation motor patterns. For the EMGs in Fig. 8, the boa constrictor used alternating bends similar to those shown in Fig. 4C, and the 45° incline required the snake to form a grip even though unlike vertical climbing some of the snake’s weight contributed to friction. Some activity of the SSP and LD on the convex side of the snake as the body straightened and slid forward occurred without simultaneous ipsilateral activity of the IC at the same longitudinal location (Fig. 8, 35–45 s). At a given longitudinal location, major bilateral activity of the IC without any synchronous activity of the SSP and LD occurred when the snake flexed ventrally to create a static grip during climbing (Fig. 8, 25–30 s), but similar bilateral activity of the IC was largely absent even when a region remained ventrally flexed as it slid forward. Finally, some low levels of bilateral activity of the SSP occurred sporadically (Fig. 8, 0–5 s and 17–20 s).

**Fig. 9:**
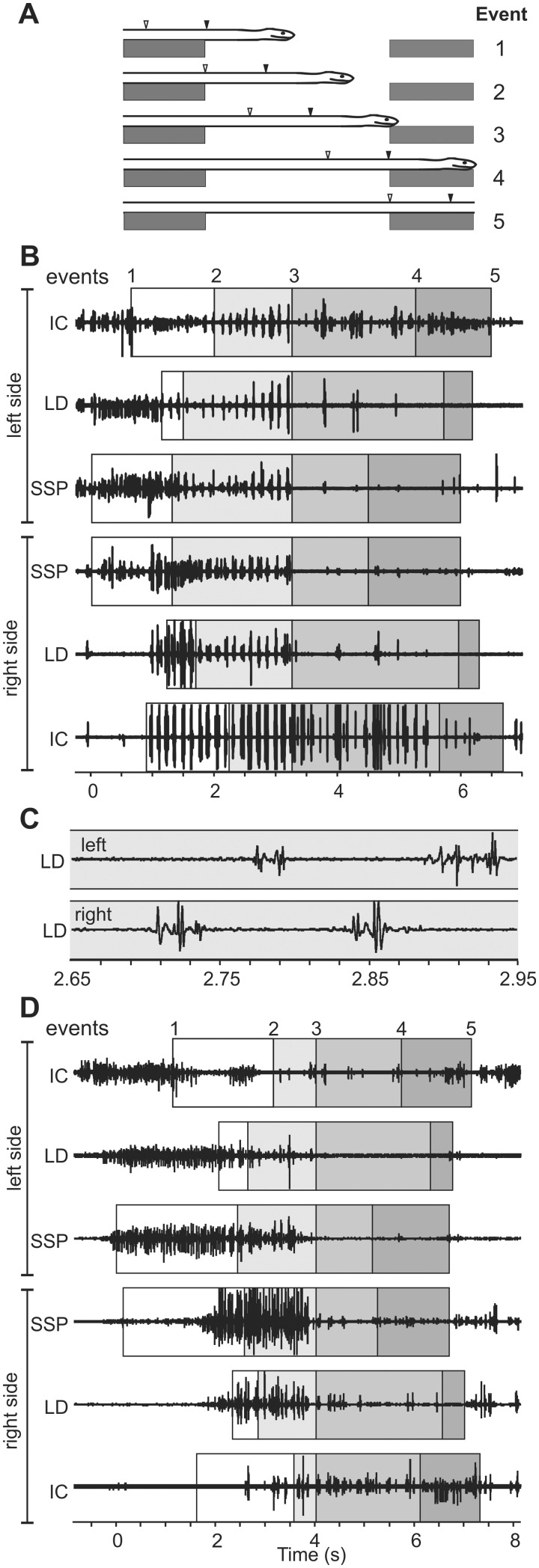
Events and EMGs of brown tree snakes (*B. irregularis*, approximate *L* = 185 cm) while bridging 43 cm gaps based on Jorgensen and Jayne (2017). (**A**) Kinematic events used to partition and analyze muscle activity. The black and white arrowheads indicate the most anterior and most posterior extent, respectively, of a particular muscle from which an EMG was obtained. (**B**) EMGs from a snake bridging a horizontal gap with a straight trajectory. (**C**) Close up of EMGs from contralateral segments of the LD during the same sequence as in (B). (**D**) EMGs from the same snake bridging a horizontal gap with a 90° turn to the left. Note the variable extent of bilateral muscle activity for contralateral pairs of homologous muscles as well as the extent to which EMG of different ipsilateral muscle segments at a given longitudinal location are simultaneous.

Occasionally, rather than using the more generalized arboreal concertina with alternate bends that grip the cylinder using ventral flexion (Figs. 4C and 7B), some snakes perform arboreal concertina with helical wrapping, in which the body resembles a coiled spring (Fig. 4D). This absence of alternating bends to the left and right in the propulsive region of the snake is distinct from all other known modes of snake locomotion that use vertebral bending for propulsion.

The orientation of the surface relative to gravity adds yet more variety to the mechanisms and modes of snake locomotion. For example, when moving vertically, the weight of the snake does not contribute to normal force and friction in the anchoring, which is unlike all types of horizontal concertina locomotion. If the weight of the snake climbing vertically is unopposed, the snake will fall down. If the weight of the snake is not balanced when moving on shallow cylindrical surfaces, the snake will topple sideways. Actively gripping cylindrical surfaces reduces both of these problems (Byrnes and Jayne 2014). However, some snakes actually take advantage of the forces from their weight by using it as a propulsive mechanism for moving down steep surfaces while holding a static posture and exerting a slight grip to control the speed of sliding (Astley and Jayne 2007).

In summary, I favor using the term “concertina” as an overarching category that encompasses four distinct modes, all of which involve periodic changes in the head-to-tail distance as well as at least a transient simultaneous occurrence of different regions of the body having static or sliding contact. I propose using the following terms to recognize four distinct types of concertina: (1) flat-surface concertina (Fig. 6A), (2) tunnel concertina (Fig. 6B), (3) arboreal concertina with alternate bends (Figs. 4C and 7B), and (4) arboreal concertina with helical wrapping (Fig. 4D).

## Bridging gaps

The complex movements involved in bridging gaps that have been studied in brown tree snakes (*Boiga irregularis*) provide several additional insights regarding the plasticity of axial motor patterns and the diversity of locomotor modes (Jorgensen and Jayne 2017). In common with many other slender species of arboreal snakes (Jayne 1982), the anterior tendon of the SSP of brown tree snakes spans more vertebrae (23) than in caenophidian locomotor generalists such as water snakes (e.g., 14 vertebrae in Fig. 2). As soon as the anterior tendon of the SSP muscle crosses the edge of a gap, high amplitude EMGs of this muscle occur on both sides of brown tree snakes (Fig. 9B, 0 s). This consistent activation of the SSP muscle for an event far from the contractile tissue differs from terrestrial locomotion where activity of the SSP correlates well with the curvature in the location of the contractile tissue but not with curvature along the far reaches of the anterior tendon of the muscular segment (Jayne 1988c). However, this initial pattern of SSP activity during gap bridging has two similarities with that of sidewinding. First, it is bilateral at a given longitudinal location. Second, it can occur in the absence of activity of ipsilateral LD and IC segments at the same longitudinal location (Fig. 9B, right side, 0.2–0.8 s).

Unlike any of the axial motor patterns previously observed for the swimming and terrestrial locomotion of snakes, during gap bridging the SSP, LD, and IC on a coarse time scale all had prolonged bilateral activity of segments at the same longitudinal location (Fig. 9B, 1–3 s). However, on a very fine time scale, left and right side bursts of activity were usually out of phase for contralateral segments of a homologous muscle (Fig. 9C).

One additional aspect of motor pattern during gap bridging was similar to arboreal concertina but absent in all types of terrestrial snake locomotion. The IC could be active without any attendant activity of the ipsilateral LD and SSP segments at the same longitudinal location (Fig. 9B, right side after 4 s). This isolated bilateral activity of IC segments at the same longitudinal location suggests that ventral tension is being actively generated to prevent sagging where the body is suspended between two points of support. By contrast, bilateral activity of the SSP causing dorsal tension appears to be the primary mechanism for supporting a suspended portion of the body when it has only one point of support during cantilevering.

When snakes bridge gaps not in a horizontal straight trajectory, additional variation in axial motor pattern occurs (Jorgensen and Jayne 2017). For example, when snakes turn left in a horizontal plane, the initial activity of the left SSP is strong, whereas that of the right side SSP is nearly absent as its anterior tendon enters the gap (Fig. 9D, 0–1 s). During turning, additional disparities between the left- and right-side activities of other homologous muscles occur that are unlike the overall pattern of similar amounts of left- and right-side activities when snakes are straight.

Some additional quantitative differences in motor pattern occur when snakes bridge gaps vertically up or down (Jorgensen and Jayne 2017). For example, when bridging upward vertical gaps, IC activity was much less than that for horizontal gaps. Snakes bridging gaps straight down also have barely any epaxial muscle activity (Jorgensen and Jayne 2017), which once again highlights how some movements can occur passively.

Bridging gaps in different directions involves substantial variation in axial motor pattern including activity of the SSP and IC that can be decoupled from that of other ipsilateral muscles in the same longitudinal location. However, we never observed substantial LD activity without some attendant simultaneous activity of either the ipsilateral SSP or ipsilateral IC at the same longitudinal location.

Collectively, these observations during gap bridging reinforce the notion that the SSP, LD, and IC are all important lateral flexors for planar movements. However, in isolation, the SSP also functions as a dorsi-flexor, and the IC in isolation functions as a ventral flexor. These data also suggest that different motor patterns would occur during terrestrial lateral undulation and arboreal lateral undulation on narrow horizontal surfaces as a result of the latter having prolonged bilateral activity of the SSP that prevents sagging (Fig. 4B).

As gap distances increase, rather than crawling slowly and continuously, some snake species may lunge, jump, or even glide (Socha 2006; Jayne et al. 2014). Perhaps these additional behaviors involve yet more variation in motor pattern and could be considered different modes.

## Future directions

A significant unresolved issue is how to best characterize some patterns of movement that do not fit precisely into any of the schemes above. For example, some snakes on sand leave drag marks between the disconnected oblique tracks that typify sidewinding (Fig. 3E). Hence, should sidewinding be defined by any combination of lateral and vertical bending with a transient period of static contact along a line that is oblique to the direction of travel, or should this mode be restricted to when the body is lifted completely off the ground? When dragging occurs, I suspect that qualitative features of the motor pattern would support a designation of sidewinding even though variable amounts of lifting and different waveforms could affect some interesting details of kinematics and mechanics (Marvi et al. 2014). Occasionally some snakes preforming terrestrial lateral undulation on planar surfaces lift parts of their body (Hu et al. 2009). However, whether this locomotion has a qualitatively different motor pattern from arboreal lateral undulation is presently unclear because activity of the SSP muscle can either prevent sagging or cause active dorsi-flexion. In light of the ability of snakes to have localized control of ventilatory movements of the ribs (Capano and Brainerd 2020), it would also be interesting to determine the extent to which different longitudinal regions of the snake could have a different locomotor motor patterns beyond those already observed for concertina locomotion and gap bridging.

Sometimes snakes on some smooth planar surfaces appear to use terrestrial lateral undulation, but all points along the body do not follow identical path because of backward slipping (resembling swimming as in Fig. 3A). Gans (1985) regarded this as a distinct mode referred to as slide pushing. However, variable amounts of backward slipping also can occur in effectively all of the types of locomotion discussed above (Figs. 1E and 6A; Marvi et al. 2014). Consequently, unless one could also find some other distinguishing qualitative difference, it would seem to me rather extreme to recognize twice as many modes as discussed above based on those with and without any backward slipping.

Another example of quantitative variation with unclear implications for the distinctness of gait also involves path following. Some arboreal concertina with alternating bends has locations of gripping and crossing over the cylinder that remain very similar while changing the length of snake between successive crossover regions propels the snake (Astley and Jayne 2009). Consequently, the differences in paths traveled and in head-to-tail distance are subtle, and the snakes appear to have bends that propagate posteriorly but do so discontinuously because of periodic stopping (Figure 4 in Astley and Jayne 2009). Because this type of movement has simultaneous sliding and static contact that typifies all concertina and the medially-directed pressing typical of arboreal concertina, little information suggests an underlying novel motor pattern exists. Given the enormous variation within the concertina group of gaits, their continued study seems likely to be fruitful.

## Parameters for identifying limbless gaits

The following 13 major traits regarding movement, friction, and muscular mechanisms can be used to categorize the different ways that snakes move (also see Supplementary Table S1). (1) Axial flexion is: absent, lateral, dorsal, or ventral. (2) Are alternating bends to the left and right present? (3) Are events (movement, bending, and EMGs) propagated? (4) What is direction of propagation? (5) Does propagation occur along the entire length of the snake? (6) The head-to-tail distance changes substantially. (7) Points along the body travel: overall similar paths, different but parallel paths, or completely different paths. (8) Contact with surfaces is: sliding, static, or both. (9) Frictional resistance is generated via: weight, pressing laterally, or pressing medially. (10) What are the muscles that are used? (11) The contralateral muscle activity at a given longitudinal location is: alternating, unilateral, or bilateral. (12) The ipsilateral muscle activity at a given longitudinal location involves synchronous or non-synchronous activity of the SSP, LD, and IC. (13) Muscle activity is: concentric, isometric, or eccentric.

## Conclusions

Recent findings, especially those arising from electromyographic studies, make a compelling case for recognizing far more distinct modes of snake locomotion than the traditional scheme of four (rectilinear, lateral undulation, sidewinding, and concertina). The terms rectilinear and sidewinding still retain their original utility as they each refer to a unique mode. By contrast and analogous to symmetric and asymmetric limbed gaits, the terms lateral undulation and concertina now seem most useful as categories of locomotion within each of which many distinct types of locomotion occur. I recognize the following five types of lateral undulation: (1) terrestrial lateral undulation, (2) forward aquatic lateral undulation, (3) backward aquatic lateral undulation, (4) lateral undulation with a ventrolateral keel, and (5) arboreal lateral undulation. Four types of concertina locomotion are: (1) flat-surface concertina, (2) tunnel concertina, (3) arboreal concertina with alternate bends, and (4) arboreal concertina with helical wrapping. Thus, I favor recognizing at least the 11 distinct modes of snake locomotion listed above that would have been traditionally considered as only four modes. If one were to include all behaviors and directions involved in crossing gaps and the variable orientations relative to gravity for movement on solid surfaces, the total number of likely distinct modes would be even greater. One should also remember that considerable quantitative variation occurs within all of the modes depending on the species of snake and the environmental conditions.

Clearly, complex systems are most easily understood by starting with the simplest cases, and this is reflected in the progression of knowledge for the locomotion of snakes. However, conclusions regarding the complexity of systems and extent to which behaviors are stereotyped can be very misleading if only the simplest cases are studied. Thus, the continued study of snake locomotion by many workers over many decades nicely illustrates the enhanced understanding of the diversity of form, function, and behavior that can emerge from building levels of complexity, in part by using the diverse behavioral repertoire of animals in nature as the ultimate guide for inquiry.

## Funding

This work was partially supported by a grant from the National Science Foundation to B.C.J [IOS 0843197]. I am especially grateful to S. A. Jayne and S. H. Jayne for paying for the publication costs as well as for more than 50 years of unwavering support for my interests in biology.

## Supplementary data

Supplementary data available at *ICB* online.

## Supplementary Material

icaa017_Supplementary_Data

## Notes

From the symposium “Long Limbless Locomotors: The Mechanics and Biology of Elongate, Limbless Vertebrate Locomotion” presented at the annual meeting of the Society for Integrative and Comparative Biology January 3–7, 2020 at Austin, Texas.
